# A dataset of factors influencing intentions for organic farming in Vietnam

**DOI:** 10.1016/j.dib.2020.106605

**Published:** 2020-11-29

**Authors:** Thi-Phuong-Linh Nguyen, Thu-Thuy Nguyen, Xuan-Hau Doan, Manh-Linh Tran, Nhat-Minh Tran, Thi-Dao Nguyen

**Affiliations:** a National Economics University, 207 Giai Phong, Hanoi, Vietnam; b Banking Academy, 12 Chua Boc, Hanoi, Vietnam

**Keywords:** Organic farming, Vietnam, Intention of farmers, Theory of planned behavior, Norm activation model

## Abstract

This paper presents a survey dataset on factors influencing farmers' intention to produce organic farming in Hanoi, Vietnam. The survey was designed based on the theoretical integration model of theory of planned behavior (TPB) and norm activation model (NAM) including 7 factors, 33 items inherited from previous studies to collect information of the respondents and 5 other items used to find out the respondent's characteristic include: gender, age, educational qualification, farming experience and farming annual income. Questionnaires were sent directly to farmers at their home or farm in October 2019. 318 valid questionnaires were collected for the study of intentions for organic farming. The dataset was obtained as a reference source for later studies on organic farming development and organic farming production intent/behavior promotion.

## Specifications Table

**Table:** 

Subject	Agriculture
Specific subject area	Organic farming, intention of farmer, theory of planned behavior, norm activation model
Type of data	Table
How data were acquired	Questionnaire
Data format	Raw Analyzed
Parameters for data collection	Participants who farmers in the Hanoi, Vietnam are practicing conventional farming, decided to participate in the survey voluntarily
Description of data collection	Data were collected through direct distribution and collection from farmers in Hanoi, Vietnam in October 2019. The data set includes 318 valid responses.
Data source location	Region: Asia Country: Vietnam Latitude and longitude: 21.028511, 105.804817
Data accessibility	Data with the article

## Value of the Data

• The dataset describes the assessment of farmers in Hanoi, Vietnam on organic farming production based on seven factors from the TPB-NAM integrated model including: intention, attitude, subject norms, perceived behavioral control, personal norm, awareness of consequences and ascription of responsibility. At the same time, the dataset also includes 5 characteristics of the respondent: gender, age, educational qualification, farming experience and farming annual income.
• The data set explores factors influencing the intentions of organic farming of farmers in Hanoi, Vietnam.
• The dataset is the source of reference for state management agencies in making policies to promote organic farming production, contributing to building a sustainable national agriculture in developing countries like Vietnam.

## 1 Data Description

Organic farming increases farmers' income, while also helping to protect environmental pollution by avoiding harmful chemicals and fertilizers [12]. Organic farming is an important tool for achieving green yields and reducing the negative effects of conventional farming by removing synthetic chemical inputs during production [1]. Therefore, developing countries like Vietnam are trying to come up with policies to promote organic farming intentions of farmers. The dataset collected farmers' opinions based on seven factors from the TPB-NAM integration model. In particular, TPB has been accepted and widely used in studies with the purpose of predicting individual intentions and behavior, empirical studies have shown the relevance of this theory in the study of farmers' intentions/behavior [3,5,6,8,11]. NAM is derived from a pro-social context and has been widely used in many studies to explain not only pro-social intentions/behavior but also pro-environmental intentions/behavior in a wide range of contexts [2,4,7,9,14].

The data set was collected through a 2-part survey: the first part explores the respondents' characteristics including: gender, age, educational qualification, farming experience and farming annual income (Table 1); the second part explores respondents' consent to statements related to factors affecting the intention to produce organic agriculture (Table 2); Table 3 shows more detailed results between the variables. It took the farmer about 20 minutes to complete the entire survey. Valuable responses were obtained in 318 questionnaires. The questionnaire and answers were shown in the supplementary files.

**Table 1:** Respondents’ characteristics

*Characteristics*	*N*	*%*	*Average value in Hanoi*
*Gender (RC1)*	318	100.00	100.00
Male	175	55.03	51.84
Female	143	44.97	48.17
*Age (RC2)*	318	100.00	100.00
From 20 to 30	114	35.85	32.84
From 31 to 40	124	38.99	37.08
From 41 to 50	51	16.04	15.93
From 51 to 60	19	5.97	7.29
Over 60	10	3.15	6.86
*Educational qualification (RC3)*	318	100.00	100.00
Haven't finished high school	60	18.87	15.07
High school	105	33.02	35.41
Intermediate	86	27.04	29.21
College/University/Postgraduate	67	21.07	20.31
*Farming experience (RC4)*	318	100.00	100.00
Under 1 year	29	9.12	7.14
From 1 to 5 years	35	11.00	13.36
From 6 to 10 years	85	26.73	24.57
From 11 to 15 years	93	29.25	30.09
Over 15 years	76	23.90	24.84
*Farming annual income (RC5)*	318	100.00	100.00
Under 5,000 USD	41	12.90	11.08
From 5,000 to 10,000 USD	54	16.98	18.56
From 10,000 to 15,000 USD	70	22.01	21.79
From 15,000 to 25,000 USD	96	30.19	29.05
Over 25,000 USD	57	17.92	19.72

**Table 2:** Descriptive results of participants’ responses

Variables		N	Min	Max	Mean	Std. Deviation
***Intention (IN) (Cronbach's Alpha = 0.782)***						

IN1	I intend to practice organic farming in my farm over the next year.	318	1	5	3.09	1.121
IN2	I will expend effort in organic farming in my farm over the next year.	318	1	5	3.60	1.178
IN3	I am planning to practice organic farming in my farm over the next year.	318	1	5	3.06	1.076
***Attitude (AT) (Cronbach's Alpha = 0.804)***						

AT1	Quality of product from organic farming is better than conventional farming.	318	1	5	3.44	1.279
AT2	Organic farming is good for farmers and the health of family members.	318	1	5	3.36	1.391
AT3	The products from organic farming are good for the consumer's health.	318	1	5	3.44	1.228
AT4	The products from organic farming are good for the environment.	318	1	5	3.29	1.324
***Subject norms (SN) (Cronbach's Alpha = 0.870)***						

SN1	Other farmer neighbors will change to organic farming.	318	1	5	3.48	1.094
SN2	Family members need the farmers to transform to organic farming.	318	1	5	3.37	1.210
SN3	Introduction and news releases from media, such as television, radio, or newspapers leads to organic farming.	318	1	5	3.45	1.160
SN4	Farmer groups on organic farming are better for exchanging information, production, and marketing.	318	1	5	3.40	1.146
SN5	Farmer groups on organic farming are better for exchanging information, production, and marketing.	318	1	5	3.46	1.125
SN6	Farmer groups on organic farming will influence others to join.	318	1	5	3.47	1.139
***Perceived behavioral control (PBC) (Cronbach's Alpha = 0.861)***						

PBC1	Farmers know the difference between organic farming and conventional farming.	318	1	5	3.52	1.240
PBC2	Farmers know the processes and techniques of organic farming.	318	1	5	3.69	1.189
PBC3	Farmers have the self-confidence to carry out organic farming.	318	1	5	3.56	1.274
PBC4	Farmers have the self-confidence to receive an organic certificate.	318	1	5	3.62	1.214
PBC5	Farmers have the self-confidence to control productivity with organic farming.	318	1	5	3.63	1.210
***Awareness of consequences (AC) (Cronbach's Alpha = 0.861)***						

AC1	Organic farming prevents pests and reduces beneficial insects.	318	1	5	2.84	1.437
AC2	Organic farming minimize soil contamination and erosion and improve fertility.	318	1	5	3.09	1.427
AC3	Organic farming help to minimize ground and surface water contaminations.	318	1	5	3.09	1.427
AC4	Organic farming prevent or reduce potential human health problems.	318	1	5	2.96	1.400
AC5	Organic farming help to improve environmental air quality.	318	1	5	3.07	1.377
***Ascription of responsibility (AR) (Cronbach's Alpha = 0.822)***						

AR1	I feel responsible for the problems of not using organic agricultural practices.	318	1	5	3.49	1.278
AR2	I provoke environmental problems if I do not use organic farming in my farm.	318	1	5	3.53	1.214
AR3	I believe that every farmer must take responsibility for organic farming.	318	1	5	3.50	1.263
AR4	All farmers are responsible for human health hazards by pesticide overuse.	318	1	5	3.64	1.228
***Personal norm (PN) (Cronbach's Alpha = 0.812)***						

PN1	I feel morally obliged to practice organic farming in my farm.	318	1	5	3.36	1.277
PN2	Organic farming is consistent with my moral principles, values, and beliefs.	318	1	5	3.35	1.212
PN3	I would feel guilty about not using organic farming in my farm.	318	1	5	3.45	1.216

**Table 3:** Correlations between variables and intention of farmers to produce organic farming.

Variable		*Intention*		
		IN1	IN2	IN3
***Respondent characteristic***				

RC1	Gender	0.011	0.009	-0.071
RC2	Age	-0.043	-0.031	-0.101
RC3	Educational qualification	-0.002	0.082	0.003
RC4	Farming experience	-0.027	-0.024	-0.109
RC5	Farming annual income	-0.054	-0.012	-0.128*
***Attitude***				

AT1	Quality of product from organic farming is better than conventional farming.	0.241⁎⁎	0.132*	0.156⁎⁎
AT2	Organic farming is good for farmers and the health of family members.	0.246⁎⁎	0.139*	0.223⁎⁎
AT3	The products from organic farming are good for the consumer's health.	0.225⁎⁎	0.193⁎⁎	0.217⁎⁎
AT4	The products from organic farming are good for the environment.	0.256⁎⁎	0.124*	0.199⁎⁎
***Subject norms***				

SN1	Other farmer neighbors will change to organic farming.	0.039	0.038	0.055
SN2	Family members need the farmers to transform to organic farming.	0.147⁎⁎	0.168⁎⁎	0.090
SN3	Introduction and news releases from media, such as television, radio, or newspapers leads to organic farming.	0.141*	0.124*	0.106
SN4	Farmer groups on organic farming are better for exchanging information, production, and marketing.	0.153⁎⁎	0.167⁎⁎	0.184⁎⁎
SN5	Farmer groups on organic farming are better for exchanging information, production, and marketing.	0.165⁎⁎	0.115*	0.182⁎⁎
SN6	Farmer groups on organic farming will influence others to join.	0.090	0.079	0.107
***Perceived behavioral control***				

PBC1	Farmers know the difference between organic farming and conventional farming.	0.222⁎⁎	0.178⁎⁎	0.273⁎⁎
PBC2	Farmers know the processes and techniques of organic farming.	0.206⁎⁎	0.183⁎⁎	0.221⁎⁎
PBC3	Farmers have the self-confidence to carry out organic farming.	0.183⁎⁎	0.174⁎⁎	0.214⁎⁎
PBC4	Farmers have the self-confidence to receive an organic certificate.	0.160⁎⁎	0.152⁎⁎	0.215⁎⁎
PBC5	Farmers have the self-confidence to control productivity with organic farming.	0.172⁎⁎	0.168⁎⁎	0.200⁎⁎
***Awareness of consequences***				

AC1	Organic farming prevents pests and reduces beneficial insects.	0.170⁎⁎	0.156⁎⁎	0.212⁎⁎
AC2	Organic farming minimize soil contamination and erosion and improve fertility.	0.170⁎⁎	0.117*	0.124*
AC3	Organic farming help to minimize ground and surface water contaminations.	0.211⁎⁎	0.223⁎⁎	0.252⁎⁎
AC4	Organic farming prevent or reduce potential human health problems.	0.147⁎⁎	0.121*	0.209⁎⁎
AC5	Organic farming help to improve environmental air quality.	0.069	0.070	0.155⁎⁎
***Ascription of responsibility***				

AR1	I feel responsible for the problems of not using organic agricultural practices.	0.220⁎⁎	0.225⁎⁎	0.175⁎⁎
AR2	I provoke environmental problems if I do not use organic farming in my farm.	0.238⁎⁎	0.244⁎⁎	0.250⁎⁎
AR3	I believe that every farmer must take responsibility for organic farming.	0.195⁎⁎	0.170⁎⁎	0.167⁎⁎
AR4	All farmers are responsible for human health hazards by pesticide overuse.	0.240⁎⁎	0.203⁎⁎	0.250⁎⁎
***Personal norm***				

PN1	I feel morally obliged to practice organic farming in my farm.	0.202⁎⁎	0.090	0.215⁎⁎
PN2	Organic farming is consistent with my moral principles, values, and beliefs.	0.239⁎⁎	0.145⁎⁎	0.280⁎⁎
PN3	I would feel guilty about not using organic farming in my farm.	0.195⁎⁎	0.164⁎⁎	0.195⁎⁎

⁎⁎ . Correlation is significant at the 0.01 level (2-tailed).

⁎ . Correlation is significant at the 0.05 level (2-tailed).

The dataset includes: the respondent's characteristics (Table 1) and seven factors: (1) intention; (2) attitude; (3) subject norms; (4) perceived behavioral control; (5) awareness of consequences; (6) ascription of responsibility; (7) personal norm (Table 2).

## 2 Experimental Design, Materials and Methods

The survey was conducted directly at the farmer's residence or farm in October 2019. The survey team received the support from Department of Science and Technology in Hanoi to list and approach the target farmers. Respondents were farmers who were practicing conventional farming in Hanoi, Vietnam. Respondents were selected at random but still ensured their representativeness in some regions that were promoting the conversion to organic farming such as Soc Son, Chuong My, Ba Vi, ... in Hanoi. Each farmer participating in the survey received a support of 2 US dollar after completing all the contents of the questionnaire which were distributed directly and collected by the survey team.

The survey team designed a survey of 38 items, of which 5 were about respondents’ characteristics, the remaining 33 items, are designed on a 5-point Likert scale (1: Strongly disagree; 2: Disagree; 3: Neutral; 4: Agree; 5: Strongly agree), focus on 7 factors: intention, attitude, subject norms, perceived behavioral control, personal norm, awareness of consequences and ascription of responsibility. All items in the survey are inherited from previous studies [10,13] and the replying is complete mandatory to ensure that the collected data does not contain missing data. The questionnaire did not use the reverse question, which was conducted directly by the survey team, with detailed observations and assisting farmers in the answer process. All responses of the respondents were imported into Excel software before importing to SPSS 22. Before the analysis, the variables were encoded (Tables 2 and 3) and the data were checked to ensure the validity of each questionnaire. After discarding invalid questionnaires, the final dataset contained 318 questionnaires.

Based on the data set, further studies can study the relationships between the factors in the TPB-NAM integration model or separate each theory to find the factors that influence intentions to produce organic farming by farmers.

## Ethics Statement

The authors kept to all ethical concerns during the data gathering process. The authors got the consent of the respondent when conducting surveys and ensured that all information was used for research purposes and was absolutely confidential.

## Declaration of Competing Interest

The authors declare that they have no known competing financial interests or personal relationships which have, or could be perceived to have, influenced the work reported in this article.

## Appendix Supplementary materials

Image, application 1

Image, application 2
