# Supplementary material for: A dataset of factors influencing intentions for organic farming in Vietnam
Source: Data Brief. 2020 Nov 29;33:106605. doi: 10.1016/j.dib.2020.106605 (PMC8129649; doi:10.1016/j.dib.2020.106605)
Supplement: Supplementary file 2 [file mmc2.docx]

**QUESTIONNAIRE**

**Factors affecting the intentions of organic farming of farmers in Vietnam**

**PART 1.** Respondents’ characteristics

***1) Gender***

| _1_ Male |
| --- |
| _2_ Female |

***2) Age***

| _1_ From 20 to 30 |
| --- |
| _2_ From 31 to 40 |
| _3_ From 41 to 50 |
| _4_ From 51 to 60 |
| _5_ Over 60 |

***3) Educational qualification***

| _1_ Haven't finished high school |
| --- |
| _2_ High school |
| _3_ Intermediate |
| _4_ College/University/Postgraduate |

***4) Farming experience***

| _1_ Under 1 year |
| --- |
| _2_ From 1 to 5 years |
| _3_ From 6 to 10 years |
| _4_ From 11 to 15 years |
| _5_ Over 15 years |

***5) Farming annual income***

| _1_ Under 5,000 USD |
| --- |
| _2_ From 5,000 to 10,000 USD |
| _3_ From 10,000 to 15,000 USD |
| _4_ From 15,000 to 25,000 USD |
| _5_ Over 25,000 USD |

**PART 2.** Please indicate your level of agreement to the following statements by adding ⌧ or 🗹 to the numbers 1 to 5. Correspondingly: 1 - strongly disagree, 2 - disagree, 3 - Neutral, 4 - agree, 5 - strongly agree.

| **Variables** | | **1** | **2** | **3** | **4** | **5** |
| --- | --- | --- | --- | --- | --- | --- |
| ***Intention (IN)*** | | | | | | |
| IN1 | I intend to practice organic farming in my farm over the next year. | ① | ② | ③ | ④ | ⑤ |
| IN2 | I will expend effort in organic farming in my farm over the next year. | ① | ② | ③ | ④ | ⑤ |
| IN3 | I am planning to practice organic farming in my farm over the next year. | ① | ② | ③ | ④ | ⑤ |
| ***Attitude (AT)*** | | | | | | |
| AT1 | Quality of product from organic farming is better than conventional farming. | ① | ② | ③ | ④ | ⑤ |
| AT2 | Organic farming is good for farmers and the health of family members. | ① | ② | ③ | ④ | ⑤ |
| AT3 | The products from organic farming are good for the consumer's health. | ① | ② | ③ | ④ | ⑤ |
| AT4 | The products from organic farming are good for the environment. | ① | ② | ③ | ④ | ⑤ |
| ***Subject norms*** | | | | | | |
| SN1 | Other farmer neighbors will change to organic farming. | ① | ② | ③ | ④ | ⑤ |
| SN2 | Family members need the farmers to transform to organic farming. | ① | ② | ③ | ④ | ⑤ |
| SN3 | Introduction and news releases from media, such as television, radio, or newspapers leads to organic farming. | ① | ② | ③ | ④ | ⑤ |
| SN4 | Farmer groups on organic farming are better for exchanging information, production, and marketing. | ① | ② | ③ | ④ | ⑤ |
| SN5 | Farmer groups on organic farming are better for exchanging information, production, and marketing. | ① | ② | ③ | ④ | ⑤ |
| SN6 | Farmer groups on organic farming will influence others to join. | ① | ② | ③ | ④ | ⑤ |
| ***Perceived behavioral control (PBC)*** | | | | | | |
| PBC1 | Farmers know the difference between organic farming and conventional farming. | ① | ② | ③ | ④ | ⑤ |
| PBC2 | Farmers know the processes and techniques of organic farming. | ① | ② | ③ | ④ | ⑤ |
| PBC3 | Farmers have the self-confidence to carry out organic farming. | ① | ② | ③ | ④ | ⑤ |
| PBC4 | Farmers have the self-confidence to receive an organic certificate. | ① | ② | ③ | ④ | ⑤ |
| PBC5 | Farmers have the self-confidence to control productivity with organic farming. | ① | ② | ③ | ④ | ⑤ |
| ***Awareness of consequences (AC)*** | | | | | | |
| AC1 | Organic farming prevents pests and reduces beneficial insects. | ① | ② | ③ | ④ | ⑤ |
| AC2 | Organic farming minimize soil contamination and erosion and improve fertility. | ① | ② | ③ | ④ | ⑤ |
| AC3 | Organic farming help to minimize ground and surface water contaminations. | ① | ② | ③ | ④ | ⑤ |
| AC4 | Organic farming prevent or reduce potential human health problems. | ① | ② | ③ | ④ | ⑤ |
| AC5 | Organic farming help to improve environmental air quality. | ① | ② | ③ | ④ | ⑤ |
| ***Ascription of responsibility (AR)*** | | | | | | |
| AR1 | I feel responsible for the problems of not using organic agricultural practices. | ① | ② | ③ | ④ | ⑤ |
| AR2 | I provoke environmental problems if I do not use organic farming in my farm. | ① | ② | ③ | ④ | ⑤ |
| AR3 | I believe that every farmer must take responsibility for organic farming. | ① | ② | ③ | ④ | ⑤ |
| AR4 | All farmers are responsible for human health hazards by pesticide overuse. | ① | ② | ③ | ④ | ⑤ |
| ***Personal norm (PN)*** | | | | | | |
| PN1 | I feel morally obliged to practice organic farming in my farm. | ① | ② | ③ | ④ | ⑤ |
| PN2 | Organic farming is consistent with my moral principles, values, and beliefs. | ① | ② | ③ | ④ | ⑤ |
| PN3 | I would feel guilty about not using organic farming in my farm. | ① | ② | ③ | ④ | ⑤ |
